# C-GATE - catalogue of genes affected by transposable elements

**DOI:** 10.1186/1759-8753-3-9

**Published:** 2012-05-23

**Authors:** Rita Rebollo, Sharareh Farivar, Dixie L Mager

**Affiliations:** 1 Terry Fox Laboratory, British Columbia Cancer Agency, 675 West 10th Avenue, Vancouver, BC, V5Z1L3, Canada; 2 Department of Medical Genetics, University of British Columbia, Vancouver, BC, Canada

**Keywords:** C-GATE, Co-option, Gene regulation, Exaptation, Transposable element

## Abstract

**Background:**

Functional regulatory sequences are present in many transposable element (TE) copies, resulting in TEs being frequently exapted by host genes. Today, many examples of TEs impacting host gene expression can be found in the literature and we believe a new catalogue of such exaptations would be useful for the field.

**Findings:**

We have established the catalogue of genes affected by transposable elements (C-GATE), which can be found at https://sites.google.com/site/tecatalog/. To date, it holds 221 cases of biologically verified TE exaptations and more than 10,000 *in silico* TE-gene partnerships. C-GATE is interactive and allows users to include missed or new TE exaptation data. C-GATE provides a graphic representation of the entire library, which may be used for future statistical analysis of TE impact on host gene expression.

**Conclusions:**

We hope C-GATE will be valuable for the TE community but also for others who have realized the role that TEs may have in their research.

## Findings

Regulation of gene expression is essential for the correct development of an organism, as it dictates where, when and how much of a gene transcript should be produced. Differences in gene expression patterns can also be associated with the divergence of species [1-3], suggesting that gene regulatory sequences are of primary importance in species evolution. In the last decades, we have learned that genes are complex units [4], harboring proximal but also distal regulatory elements and very often capable of producing more than one transcript through multiple promoters, alternative splicing and cryptic polyadenylation sites. There are different mechanisms that may be responsible for the origin and evolution of gene regulatory sequences: *de novo* synthesis; transposition (ready-to-use regulatory elements brought by sequences and spread throughout the genome); co-option of existing regulatory sequences into new functions; and mutations, deletions and duplications within existing regulatory sequences. Transposable elements (TEs) are DNA sequences able to jump throughout the genome and increase in copy number. Through transposition, TEs have a direct impact on genome size and therefore increase the genetic repertoire, which in consequence may be the target of *de novo* evolution. Furthermore, TEs have ready-to-use regulatory sequences that may be exapted as promoters and enhancers, binding sites, splice sites, polyadenylation signals, insulators and termination sites. Since some TE families are species-specific, TEs could also account for species-specific regulatory sequences. In agreement with this, the number of examples of TEs impacting host gene expression is increasing in the literature, particularly with the advent of genome-wide next-generation sequencing technologies. For instance, several groups have found transcription start sites in mammals to be frequently positioned within TE sequences [5-7]. While the search for conserved regulatory elements is able to demonstrate ancient waves of TE insertions that have contributed to regulatory sites [8,9], comparisons between species-specific regulatory sequences show that recent TE transpositions have also donated new regulatory elements to different species [9,10]. Interestingly, TE families and copies may colonize different species genomes but act as equivalent gene regulatory sequences, as observed with the convergent evolution of *NAIP* promoters in mouse and human for example [11].

Because of the large amount of data on TE exaptations present in the literature today, including lists in large non-ergonomic supplementary tables, we have decided to create an online database that catalogues published examples of TE exaptations allowing for researchers to easily browse the data. The catalogue of genes affected by transposable elements (C-GATE) is available at https://sites.google.com/site/tecatalog/. We thank the efforts of others to catalogue such exaptations, in particular Brosius [12] and Makalowski [13] and other groups [7,14]. While these data-sets are informative, those cited in the work of Brosius and of Makalowski are out-of-date, and none are interactive and therefore do not allow for user input and updates. We have intentionally designed C-GATE to be interactive so that any missed or new examples of TEs influencing host gene expression can be easily added by any investigator in the field. All submitted new exaptation events will be analyzed for integrity and significance before being added to C-GATE. Furthermore, the catalogue can be filtered and is searchable, making it easy to take advantage of the entire data set. Users are also able to download the whole catalogue.

It is important to note that another currently active online catalogue of TE exaptations exists, TranspoGene [15], which is based on an *in silico* analysis of seven vertebrates and invertebrates species. While TranspoGene remains an interesting resource for genome-wide impact of TE copies, it does not contain TE exaptations described in the literature, but solely examples observed by the authors, at the time of their analysis. C-GATE contains all the data on exaptation available at TranspoGene but also aims to include all exaptation events described in the literature. Furthermore, C-GATE contains two types of data, a general C-GATE data-set that holds only biologically confirmed and published TE exaptation examples, and a pC-GATE that holds data-sets of putative TE exaptations from ESTs, chromatin immunoprecipitation sequencing and other published *in silico-*only analyses. In order to be part of C-GATE, a TE exaptation needs to be observed in wild-type species, not only in mutants or cancer cell lines. TEs impacting specific inbred strains, as *Drosophila melanogaster P* element collections, or mouse endogenous retrovirus insertions are considered as genetic mutations and not true TE exaptations. C-GATE also does not include open reading frame domestication, as described for *syncytin* genes for instance and often reviewed in the literature [16,17]. Depending on the usage of C-GATE and the demand of the users, a future upgrade could include such domestication events and address other user concerns.

**Figure 1:**
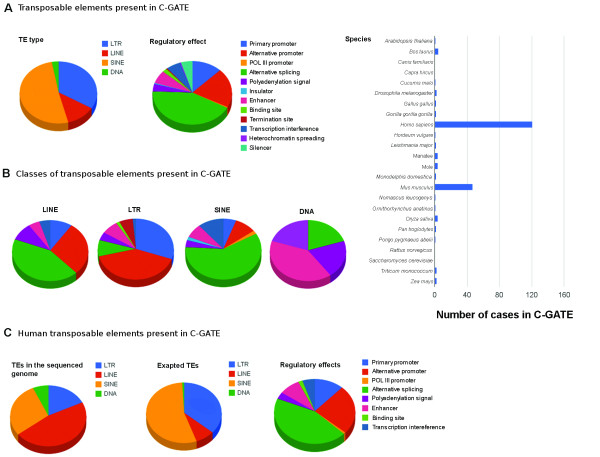
**Graphic representation of C-GATE at time of publication. (A) C-GATE general graphs.** Pie charts depicting the proportion of TE types (LTR, LINE, SINE, DNA), their regulatory effects on host genes and a bar chart showing species concerned for all examples found in the general C-GATE (biologically confirmed cases). **(B)** Graphs per TE type present in the C-GATE. Pie charts of regulatory impact of TEs on host genes, separated by TE types. The legend is the same as panel A, regulatory elements. **(C)***Homo sapiens* exapted TEs. Graphic representation of all TE types and their regulatory effects in the human genome. The first pie chart also depicts the proportion of TEs present in the human genome (100% is equal to all TE types in the genome) based on the published sequenced genome [20]. In order to view the updated graphs, please go to the C-GATE website http://sites.google.com/site/tecatalog/. C-GATE: catalogue of genes affected by transposable elements; TE: transposable elements.

C-GATE is formatted as shown in Table 1, and each user can either upload an example through an online form or multiple examples by downloading a table and submitting it in the C-GATE forum. A ‘comment’ section allows for more descriptive information regarding the publication, facilitating user comprehension of each case hosted within the catalogue. The website also holds graphic visualization of the general C-GATE data-set that is automatically updated with every new entry uploaded (Figure 1). Such graphic representations might be useful in the future to access exaptation frequency between TEs. Today, the catalogue shows a biased representation of human and mouse examples, which we hope will decrease with usage. For instance, almost 4,000 human genes are present in both C-GATE and pC-GATE. At the time of publication C-GATE, although incomplete, holds 221 cases previously described in the literature and our pC-GATE harbors more than 10,000 examples. We reinforce the notion that C-GATE is not complete and many already published TE exaptation examples are still to be included and we hope users will participate in this task. We want this database to help researchers obtain information on particular TE sequences or determine if their gene of interest is controlled by a TE copy. We invite researchers to discuss the catalogue on the forum present in C-GATE and we also expect many new examples of exapted TEs to be inserted by the users in the near future.

**Table 1:** Examples of C-GATE

Species *	Type *	Family	Subfamily	Regulatory effect *	Comments *	Gene *	Gene regulatory networks	Environment response?	Date of publication *	Reference (1) *	Reference (2)
*Latin name*	*LTR, LINE, SINE, DNA*	*TE family*	*TE sub-family*	*How does the TE impact the gene?*	*A clear summary of the publication and/or details on the exaptation event.*	*Gene with the TE exaptation*	*Is the TE or gene part of a regulatory network?*	*Is the exaptation a response to environment changes?*		*Reference with a link to the journal*	*Second reference if necessary*
*Homo sapiens*	LTR	ERV-1	HERV-9	Alternative promoter	12% of NAIP total expression in testis is due to a LTR9.	NAIP			2007	Romanish et al. (2007) PloS Genetics [11]	
*Homo sapiens*	LTR	ERV-3	Mer74C	Primary promoter	Bioinformatic analysis of human and mouse RefSeq UTRs. CA1 is transcribed through two promoters one of which is a LTR copy, present in both human and mouse that confers erythroid specific expression in both species. Chimeras were confirmed through bibliography or RT PCR. Coordinates are from hg18.	CA1			2003	Van de Lagemaat et al. (2003) Trends Genet [18]	Piriyapongsa et al. (2007) BMC Genomics [19]

* Mandatory field.

Note that columns containing coordinates of the TE, its position relative to the gene and any influence on phenotype have been omitted for clarity but are present in C-GATE.

## Abbreviations

C-GATE: catalogue of genes affected by transposable elements; EST: expression sequence tag; pC-GATE: putative C-GATE; TE: transposable elements.

## Competing interests

The authors declare that they have no competing interests.

## Authors’ contributions

RR designed the website. RR, SF and DM included cases to the website. RR and DM wrote the paper. All authors read and approved the final manuscript.
